# TAS0314, a novel multi-epitope long peptide vaccine, showed synergistic antitumor immunity with PD-1/PD-L1 blockade in HLA-A*2402 mice

**DOI:** 10.1038/s41598-020-74187-6

**Published:** 2020-10-14

**Authors:** Yuki Tanaka, Hiroshi Wada, Risa Goto, Toshihiro Osada, Keisuke Yamamura, Satoshi Fukaya, Atsushi Shimizu, Mitsuru Okubo, Kazuhisa Minamiguchi, Koichi Ikizawa, Eiji Sasaki, Teruhiro Utsugi

**Affiliations:** Discovery and Preclinical Research Division, Taiho Pharmaceutical Co. Ltd., Tsukuba, Ibaraki Japan

**Keywords:** Peptide vaccines, Tumour immunology

## Abstract

Cancer peptide vaccines are a promising cancer immunotherapy that can induce cancer-specific cytotoxic T lymphocytes (CTLs) in tumors. However, recent clinical trials of cancer vaccines have revealed that the efficacy of the vaccines is limited. Targeting single antigens and vaccination with short peptides are partly the cause of the poor clinical outcomes. We synthesized a novel multi-epitope long peptide, TAS0314, which induced multiple epitope-specific CTLs in *HLA* knock-in mice. It also showed superior epitope-specific CTL induction and antitumor activity. We also established a combination treatment model of vaccination with PD-1/PD-L1 blockade in HLA-A*2402 knock-in mice, and it showed a synergistic antitumor effect with TAS0314. Thus, our data indicated that TAS0314 treatment, especially in combination with PD-1/PD-L1 blockade, is a promising therapeutic candidate for cancer immunotherapy.

## Introduction

Cancer vaccines that can induce a tumor-specific immune response without serious adverse events are attractive as a therapy for cancer. Numerous cancer antigens have been discovered and developed as peptide-, DNA-, or RNA-based cancer vaccines^1^. Peptide vaccines have been developed since the 1990s, but only limited therapeutic effects have been shown in the clinical setting.

EGFR^2^, Lck^3–5^, MRP3^6^, SART2^7^, SART3^8–10^, PTHrP^11^, TMEM189 (also referred as UBE2V)^12^, and WHSC2^12^ were identified as common cancer antigens among various cancers, and epitope peptides from these antigens efficiently induced HLA-restricted cytotoxic T lymphocyte (CTL) responses in clinical trials^13,14^. However, peptide vaccines using these antigens did not prolong the overall survival of patients in phase III clinical trials^15^. Many studies on how to overcome these limited therapeutic effects of peptide vaccines have been carried out, and several problems regarding cancer peptide vaccines have been revealed.

First, peptide vaccine-induced specific CTLs fail to efficiently accumulate in tumors^16^. Incomplete Freund’s adjuvant is widely used as an adjuvant for cancer peptide vaccines. However, a recent study revealed that epitope peptides emulsified with incomplete Freund’s adjuvant induced the accumulation of epitope-specific CTLs at the injection site, and failed to induce efficient antitumor effects. However, a long peptide did not induce accumulation at the injection site, and could induce an efficient antitumor effect.

Second, immune checkpoint molecules on CTLs induce exhaustion. PD-1 is a molecule that is expressed on exhausted T cells, and it interacts with PD-L1 in the tumor microenvironment^17^. PD-1/PD-L1 interaction suppresses cytokine production and proliferation, and leads to T cell dysfunction^18^. To enhance T cell immunity, PD-1/PD-L1 blockades have been developed, and they showed strong clinical efficacy in various types of cancers^19–23^. It has been reported that vaccine-induced CTLs also express PD-1, and the combination of a peptide vaccine with anti-PD-1 antibody synergistically induced antitumor effects in a mouse tumor model^24^. Therefore, PD-1/PD-L1 blockade is expected to augment the efficacy of cancer vaccines in clinical trials.

Third, cancers can evade vaccine-induced CTLs via tumor antigen loss. The CTLs induced by peptide vaccines mediate cytotoxicity only against antigen-expressing tumors. In some tumors, the expression of tumor antigens is altered by epigenetic regulation or mutation, enabling the tumors to evade the immune system^25^. For example, in the phase II clinical trial of the glypican-3 peptide vaccine, tumors that relapsed after vaccination showed the loss of glypican-3 antigen^26^. Immunization with multiple epitopes is one of the ways to overcome the immune evasion of tumors, and would enhance the antitumor effect^27^. Therefore, it is possible to enhance the efficacy of anti-cancer peptide vaccines to overcome these three problems.

To overcome the issues of existing antitumor peptide vaccines, we designed and developed a novel multi-epitope long peptide vaccine, TAS0314, which contains four epitopes from SART2 and SART3. The aim of this study was to demonstrate the immunological characteristics of TAS0314 in vitro and in vivo as an antitumor peptide vaccine. We also evaluated the benefit of combination treatment with PD-1/PD-L1 blockade using recently established HLA-A*2402 mice.

## Results

### TAS0314 induced multiple epitope-specific CTLs

To evaluate the efficacy of a multi-epitope long peptide vaccine, we synthesized a novel peptide, TAS0314, which contains four epitopes from SART2 and SART3 linked with an arginine dimer.

**Figure 1:**
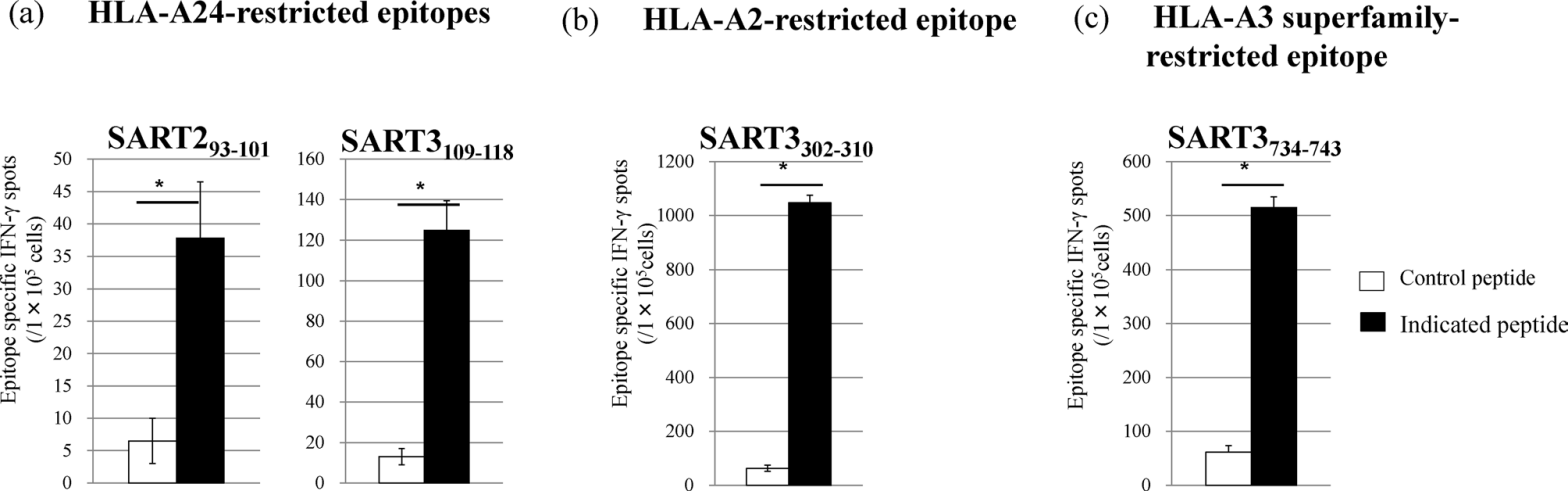
CTL induction by TAS0314 vaccination in various HLA-KI mice. Mice (n = 10) were treated with TAS0314 (300 μg/each peptide) once a week. One week after the last immunization, draining lymph node cells were isolated, and pooled lymphocytes were cultured for 8 days with epitope peptide, IL-15, and IL-21. Epitope-specific CTLs were evaluated with an IFN-γ ELISPOT assay (four wells/peptide). (**a**) HLA-A24-restricted epitope-specific CTL induction in HLA-A*2402 KI mice. (**b**) HLA-A2-restricted epitope-specific CTL induction in HLA-A*0201 KI mice. (**c**) HLA-A3 superfamily-restricted epitope-specific CTL induction in HLA-A*3101 KI mice. Data represent the mean ± standard deviation (n = 4). *p < 0.05 using a two-tailed Student’s *t* test.

First, we evaluated the CTL induction ability of TAS0314 by using *HLA* knock-in (KI) mice that were established previously^28^. The CTL induction ability of HLA-A*2402-restricted epitopes SART2_93–101_ and SART3_109–118_ was determined by using HLA-A*2402 KI mice (Fig. 1a). Lymph node cells from immunized mice produced interferon (IFN)-γ after stimulation from the respective epitope peptides, indicating that TAS0314 can induce HLA-A*2402-restricted epitope peptide-specific CTLs.

We also confirmed the CTL induction ability of HLA-A*0201-restricted epitope SART3_302-310_ as well as HLA-A3 superfamily-restricted epitope SART3_734–742_ using HLA-A*0201 KI mice or HLA-A*3101 KI mice (Fig. 1b,c).

### TAS0314 efficiently induced memory CTLs

**Figure 2:**
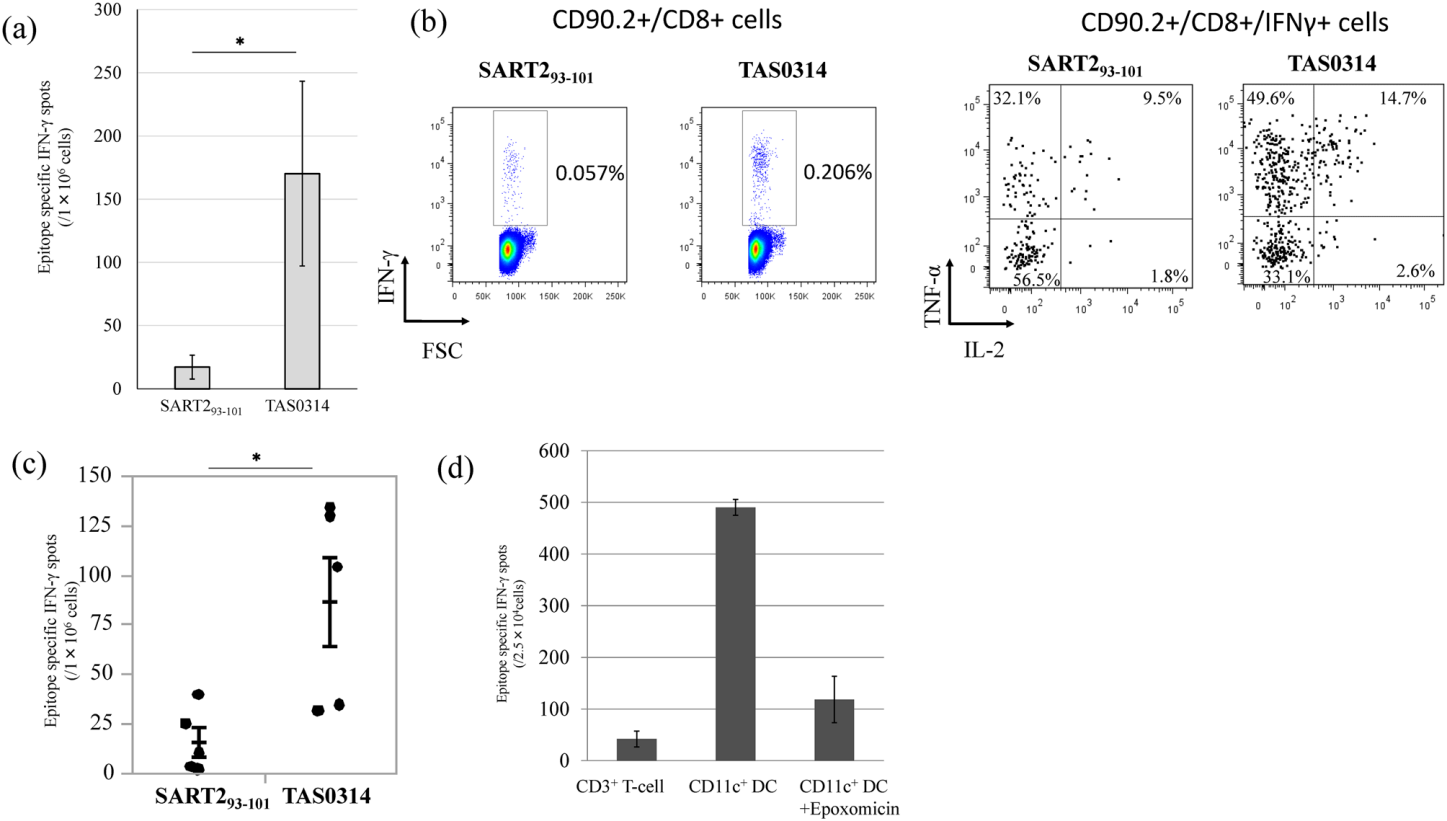
Comparison of the SART2_93–101_-specific CTL induction and its function between short (SART2_93–101_) and long (TAS0314) peptides. (**a**) Comparison of the frequency of SART2_93–101_-specific CTLs. HLA-*A2402 KI mice (n = 10/group) were vaccinated with TAS0314 (100 µg) or SART2_93–101_ peptide (21 µg, equivalent molar mass to TAS0314). One week after the last immunization, SART2_93–101_-specific IFN-γ production from the lymphocytes of each mouse was evaluated with an IFN-γ ELISPOT assay. Data represent the mean ± standard error (n = 10). (**b**) Cytokine multi-functionality of SART2_93–101_-specific CTLs. Peripheral blood mononuclear cells were prepared from immunized mice (n = 10/group) and stimulated overnight with SART2_93–101_ peptide (10 μM). The production of IFN-γ, TNF-α, and IL-2 was analyzed in CD90.2^+^/CD8^+^ cells. (**c**) Comparison of the frequency of SART2_93–101_-specific CTLs in the prime-boost vaccination condition. HLA-*A2402 KI mice (n = 5/group) were vaccinated three times at weekly intervals with TAS0314 (100 µg) or SART2_93–101_ peptide (21 µg). One hundred and forty-eight days after the last immunization, all mice were immunized with SART2_93–101_ peptide (21 µg). Data represent the mean ± standard error (n = 5). (**d**) Evaluation of the antigen presentation of TAS0314. SART3_302–310_ epitope-specific CTLs were cultured with TAS0314-pulsed CD3^+^ T cells, CD11c^+^ DCs, or epoxomicin-treated CD11c^+^ DCs. Data represent the mean ± standard deviation (n = 4). *p < 0.05 using a two-tailed Student’s *t* test.

We compared the frequencies of epitope-specific CTLs induced by TAS0314 and SART2_93–101_ peptide, which makes up TAS0314, at an equivalent molar mass. Interestingly, TAS0314 induced a higher frequency of SART2_93–101_-specific CTLs than SART2_93–101_ peptide (Fig. 2a). In order to clarify the difference in CTL function due to immunization with TAS0314, we focused on the cytokine production capacity of the CTLs in blood. TAS0314 markedly increased the population of IFN-γ^+^/tumor necrosis factor (TNF)-α^+^ double-positive CTLs and IFN-γ^+^/TNF-α^+^/interleukin (IL)-2^+^ triple-positive multifunctional CTLs in blood when compared to SART2_93–101_ peptide vaccination (Fig. 2b).

To evaluate whether the CTLs induced by TAS0314 can exert long-term surveillance, we examined the recall response against SART2_93–101_ peptide. Mice were immunized with TAS0314 or SART2_93–101_ peptide and were boosted with SART2_93–101_ 148 days after the last immunization (Fig. 2c). Mice immunized with TAS0314 had significantly increased numbers of specific CTLs after boosting when compared to those immunized with SART2_93–101_.

To evaluate the mechanism of antigen presentation for TAS0314, we examined the antigen presentation of TAS0314 by professional antigen-presenting cells (DCs) and non-professional antigen-presenting cells (T cells). As shown in Fig. 2d, TAS0314-pulsed CD11c^+^ DCs efficiently activated CTLs while TAS0314-pulsed T cells exhibited a significantly lower IFN-γ response. In addition, the CTL activation by TAS0314-pulsed DCs was significantly decreased by the proteasome inhibitor epoxomicin^29^.

### CTLs induced by TAS0314 killed antigen-expressing cells in vitro

**Figure 3:**
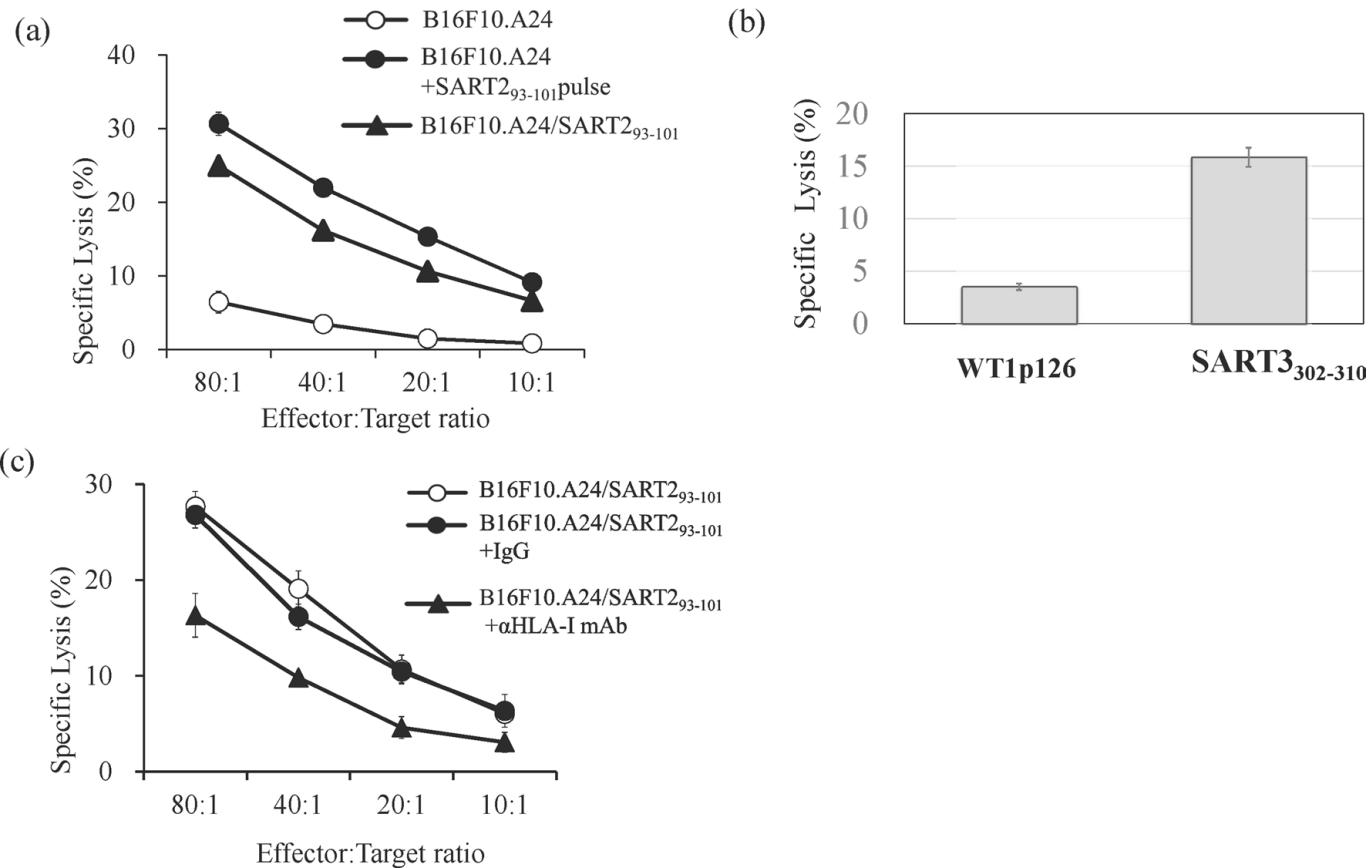
Antigen-specific cytotoxic activity of SART2_93–101_-specific CTL induced by TAS0314. (**a**) Evaluation of SART2_93–101_ epitope-specific cytotoxicity. SART2_93–101_-specific CTLs were cultured with ^51^Cr-labeled B16F10.A24, SART2_93–101_ peptide-pulsed B16F10.A24, or B16F10.A24/SART2_93–101_ cells. (**b**) Evaluation of SART3_302–310_ epitope-specific cytotoxicity. SART3_302–310_-specific CTLs were cultured with ^51^Cr-labeled SART3_302–310_ peptide or WT1 p126 peptide-pulsed T2 cells. Effector: Target ratio was 80:1. (**c**) HLA neutralization antibody blocked SART2_93–101_-specific cytotoxicity. SART2_93–101_-specific CTLs were cultured with ^51^Cr-labeled B16F10.A24/SART2_93–101_ cells in the presence of anti-HLA-ABC blocking antibody or isotype control antibody (20 µg/mL). Data represent the mean ± standard deviation (n = 3).

To confirm whether the TAS0314-induced CTLs had cytotoxicity against tumor cells, epitope-specific cytotoxicity was evaluated using a 51-chromium (^51^Cr) cytotoxicity assay. SART2_93–101_ peptide-specific CTLs from TAS0314-immunized mice were expanded for a week, then co-cultured with B16F10 cells expressing HLA-A*2402 (B16F10.A24) and epitope peptide. Strong cytotoxicity was observed against B16F10.A24 cells only when SART2_93–101_ peptide was pulsed (Fig. 3a).

SART3_302–310_ epitope-specific cytotoxicity was also evaluated using T2, an HLA-A2-positive human cell line. The cultured SART3_302–310_ peptide-specific CTLs showed cytotoxicity against the peptide-pulsed T2 cells (Fig. 3b).

We measured the cytotoxicity against a tumor cell line, B16F10.A24/SART2_93–101_ cells, that overexpressed both HLA-A*2402 and SART2_93–101_ peptide. As expected, the CTLs induced by TAS0314 displayed strong cytotoxicity against B16F10.A24/SART2_93–101_ cells (Fig. 3a). The addition of HLA-neutralizing antibody resulted in a 40% reduction of cytotoxicity (Fig. 3c). These data revealed that the CTLs induced by TAS0314 can recognize epitopes in an HLA-dependent manner.

### Antitumor effect of TAS0314 against B16F10.A24/SART2_93–101_ tumor cells

**Figure 4:**
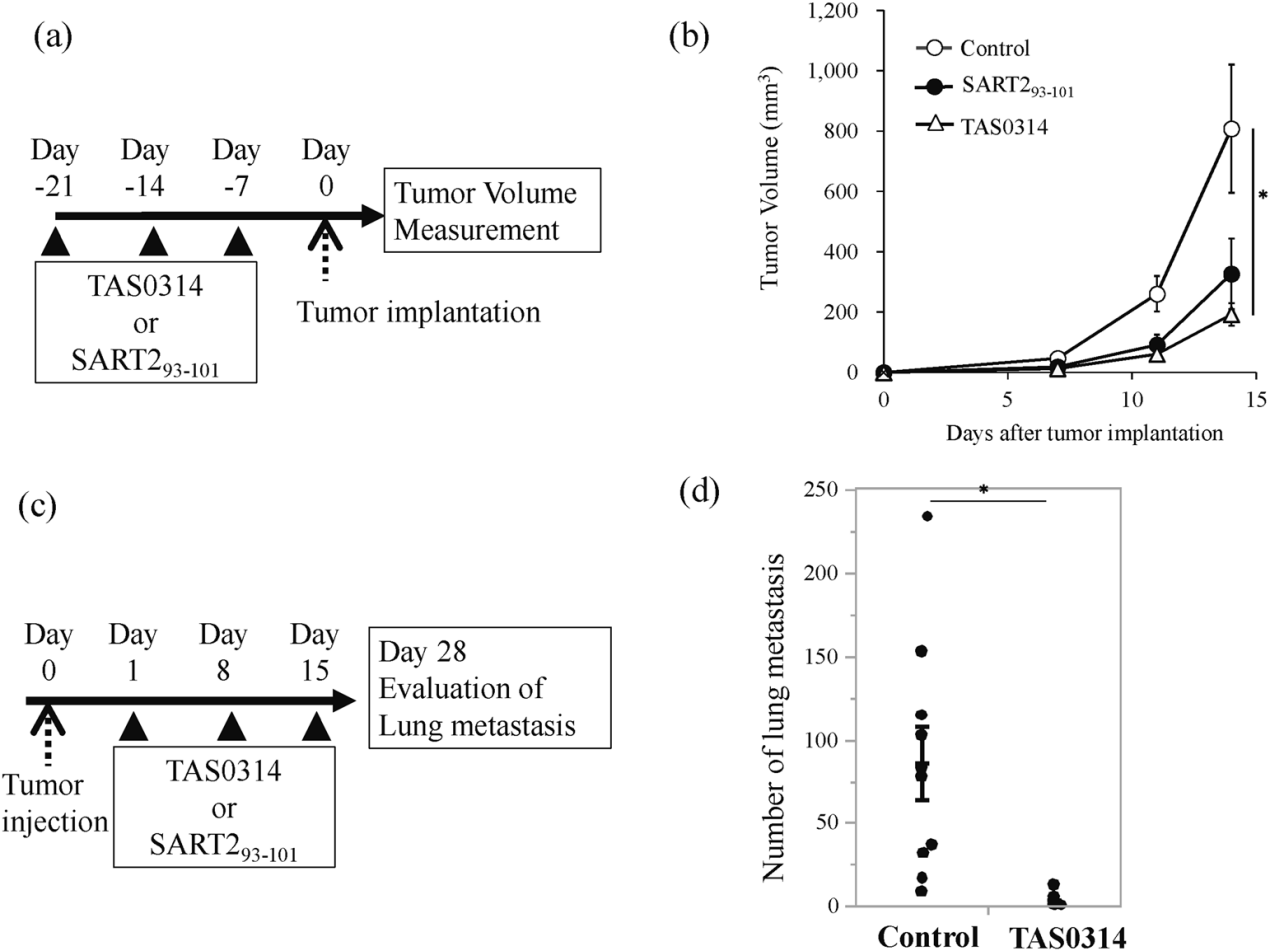
Antitumor activity of TAS0314 against syngeneic tumors. (**a**,**b**) Antitumor activity of prophylactic treatment with TAS0314. HLA-A*2402 KI mice (n = 15/group) were vaccinated with TAS0314 (100 µg) or SART2_93–101_ peptide (21 µg). One week after the last immunization, B16F10.A24/SART2_93–101_ cells were inoculated into the right flank of the mice. (**a**) Experimental scheme. (**b**) Plot of the tumor growth. Results are expressed as the mean ± standard error. (**c**,**d**) Antitumor activity of TAS0314 against lung metastasis. HLA-A*2402 KI mice (n = 10/group) were injected with B16F10.A24/SART2_93–101_ cells, then immunized with TAS0314 (300 µg). Twenty-eight days after implantation, the number of lung metastases was counted. (**c**) Experimental scheme. (**d**) Evaluation of lung metastasis. Results are expressed as the mean ± standard error. *p < 0.05 using a two-tailed Student’s *t* test.

We evaluated the antitumor activity against subcutaneous B16F10.A24/SART2_93–101_ tumors in a syngeneic mouse model. To compare the efficacy, mice were immunized with SART2_93–101_ peptide or TAS0314 at a molar-equivalent dose (Fig. 4a). At 18 days after tumor implantation, TAS0314 significantly reduced the tumor volume by 70.25% when compared to the control. SART2_93–101_ peptide only reduced the tumor volume by 47.7%, and did not show any significant difference from the control group (Fig. 4b). These data suggested that TAS0314 induced better antitumor activity than SART2_93–101_.

We also evaluated antitumor activity with a syngeneic lung metastasis model of B16F10.A24/SART2_93–101_ cells (Fig. 4c). The number of metastatic lung nodules was significantly decreased in the TAS0314-treated group when compared to the control group (Fig. 4d). TAS0314 showed antitumor activity not only in the subcutaneous model, but also in the metastasis model.

### TAS0314 and PD-1/PD-L1 blockade synergistically suppressed tumor growth

**Figure 5:**
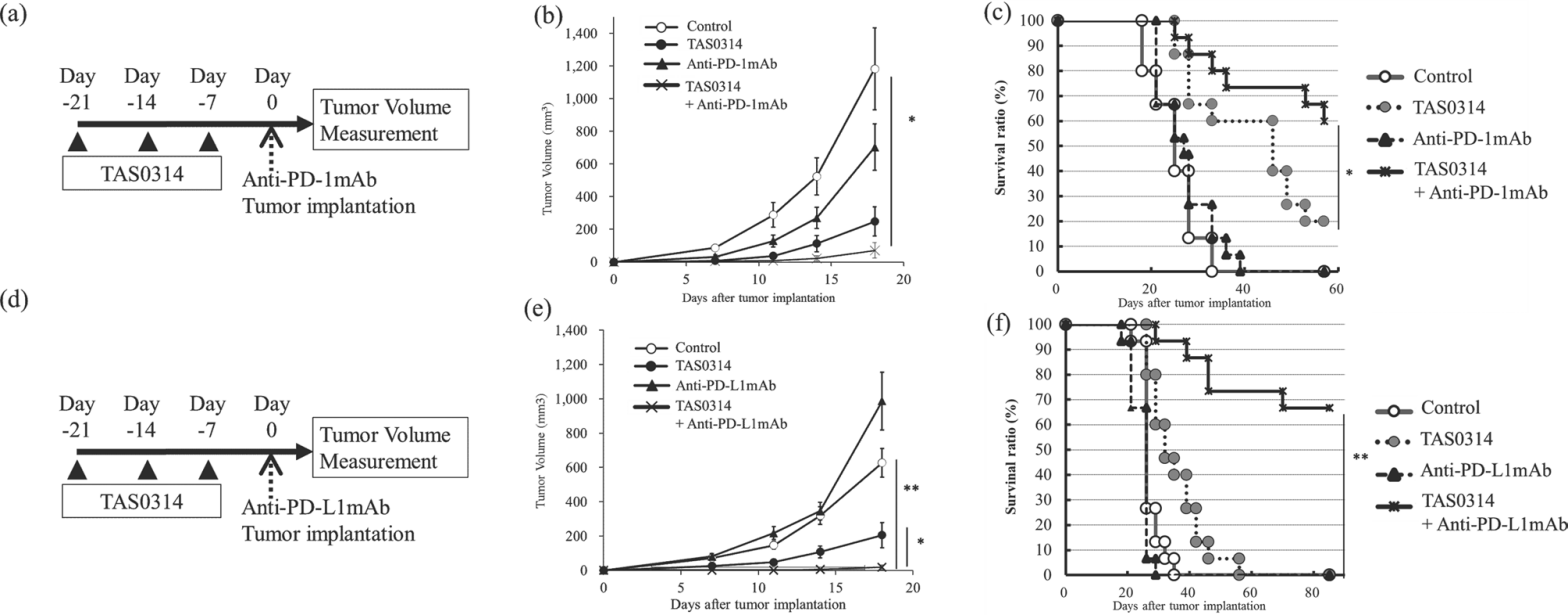
The synergistic antitumor effect of TAS0314 and PD-1/PD-L1 blockade. (**a**–**c**) Combination therapy of TAS0314 and anti-PD-1 mAb. HLA-A*2402 KI mice (n = 15/group) were treated with TAS0314 (300 µg/each) and/or anti-PD-1 mAb (100 µg), then implanted with B16F10.A24/SART2_93–101_ tumors. (**a**) Experimental scheme. (**b**) Tumor growth curves. (**c**) Survival curves. (**d**–**f**) Combination therapy of TAS0314 and anti-PD-L1 mAb. HLA-A*2402 KI mice (n = 15/group) were treated with TAS0314 (300 µg/each) and/or anti-PD-L1 mAb (200 µg), then implanted with B16F10.A24/SART2_93–101_ tumors. (**d**) Experimental scheme. (**e**) Tumor growth curves. (**f**) Survival curves. Results are expressed as the mean ± standard error. Mice were monitored until the tumor volume had reached 2000 mm^3^ or until death. Tumor volume was assessed by a two-tailed Student’s *t* test, and survival was evaluated by a log-rank test. *p < 0.05; **p < 0.001.

We evaluated the synergistic antitumor activity of TAS0314 and anti-PD-1 antibody against B16F10.A24/SART2_93–101_ tumors (Fig. 5a). Anti-PD1 antibody treatment partially suppressed tumor growth, while TAS0314 treatment significantly suppressed tumor growth (Fig. 5b). Furthermore, the combined therapy of TAS0314 and anti-PD-1 antibody dramatically suppressed tumor growth when compared to monotherapy.

We also evaluated the survival for 57 days. Anti-PD-1 antibody did not prolong the survival when compared to the control group (Fig. 5c). Combined treatment with TAS0314 and anti-PD-1 antibody significantly prolonged the survival when compared to the anti-PD-1 antibody alone (p < 0.001) or TAS0314 alone (p < 0.05). At 57 days after tumor implantation, all mice in the control group and the anti-PD-1 antibody-treated group were dead. In contrast, 13.33% of the mice were tumor-free in the TAS0314-treated group, and 53.33% of the mice were tumor-free in the group treated with the combination of TAS0314 and anti-PD-1 antibody.

We also evaluated the antitumor activity of TAS0314 in combination with anti-PD-L1 antibody (Fig. 5d). The combination of TAS0314 and anti-PD-L1 antibody also synergistically inhibited tumor growth when compared to monotherapy (Fig. 5e). Combination therapy with anti-PD-L1 antibody dramatically prolonged the survival (Fig. 5f). Eighty-five days after tumor implantation, 66.67% of the mice treated with the combination of TAS0314 and anti-PD-L1 antibody were tumor-free. In contrast, no mice were alive in the other groups.

These data strongly suggested that the combination treatment of TAS0314 and PD-1/PD-L1 blockade has synergistic antitumor effects.

### Combination of TAS0314 and anti-PD-1 antibody increased the number of epitope-specific CTLs in tumors

**Figure 6:**
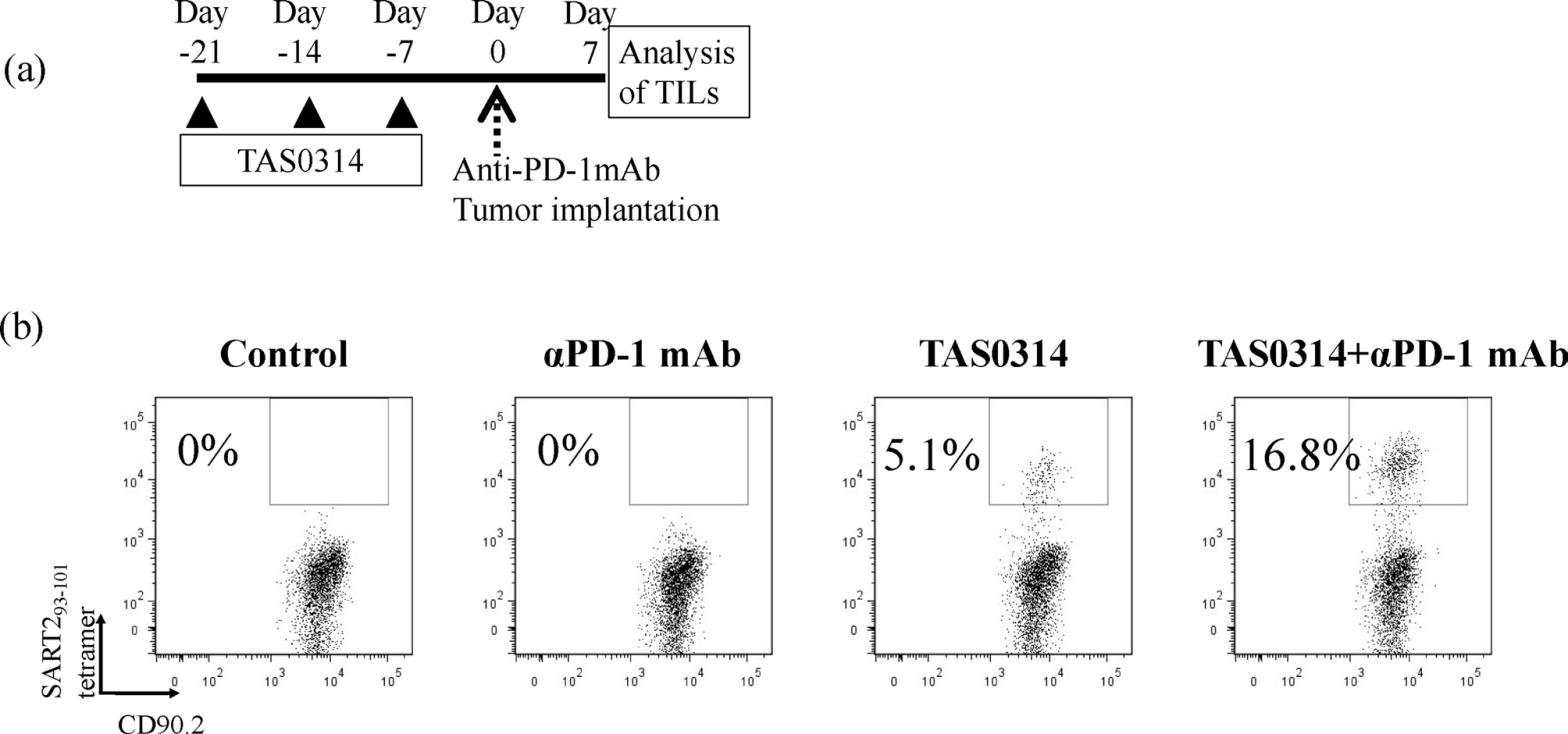
Comparison of SART2-specific CTL infiltration into tumors. HLA-A*2402 KI mice (control and anti-PD-1 mAb: n = 5/group; TAS0314 and TAS0314 + anti-PD-1 mAb: n = 10/group) were treated with TAS0314 (25 μg/each) and/or anti-PD-1 mAb (100 µg), then implanted with B16F10.A24/SART2_93–101_ tumors. Seven days after tumor implantation, the number of SART2_93–101_ epitope-specific CTLs in tumors was analyzed by flow cytometry. (**a**) Experimental scheme. (**b**) Comparison of SART2-specific CTL infiltration.

To analyze the mechanism of the synergistic effect, we measured SART2_93–101_ epitope-specific T cells in tumors using the SART2_93–101_–HLA-A24 tetramer (Fig. 6). A number of tetramer-positive T cells was detected after immunization with TAS0314, whereas no SART2-specific CTL infiltration was detected in the vehicle-immunized groups. Surprisingly, the number increased approximately three-fold in the group treated with the combination treatment of TAS0314 and anti-PD-1 antibody. Increases in the number of epitope-specific CTLs in tumors would contribute to the synergistic antitumor activity of TAS0314 and anti-PD-1 antibody.

## Discussion

Numerous clinical trials have been conducted, but the efficacy of cancer vaccines has been limited^30,31^. To develop useful cancer peptide vaccines, their efficacy must be improved.

We developed a novel multivalent long peptide vaccine, TAS0314, and evaluated its CTL induction ability, antitumor effect in monotherapy, and its antitumor effect in combination with anti-PD-1 antibody. We previously reported that the induction of epitope-specific CTLs is affected by the position of the epitope in a long peptide^32^. We evaluated and screened numerous peptides to obtain suitable long peptides with efficient CTL induction ability by changing the linkage order of four epitopes. Finally, we designed TAS0314, which could efficiently induce epitope-specific CTL induction. Indeed, TAS0314 showed superior CTL induction and antitumor effects when compared to short epitope peptide vaccination.

Synthetic long peptide vaccines that were efficiently processed by DCs and induced robust CD8^+^ T cell responses have been reported^33^. In our study, we confirmed that TAS0314 epitopes were presented by DCs in a proteasome-dependent pathway. In contrast, non-professional antigen-presenting cells, such as T cells, showed little antigen presentation of the epitopes in TAS0314. Antigen presentation by non-professional antigen-presenting cells induces the exhaustion of CTLs, and exhausted T cells show loss of multifunctional cytokine production^34^. Indeed, we obtained similar data showing that T cells induced by short epitope immunization had a smaller population of multifunctional (IFN-γ^+^/TNF-α^+^/IL-2^+^, IFN-γ^+^/TNF-α^+^) CTLs when compared to those induced by TAS0314. We considered that TAS0314 antigen presentation via DCs may contribute to the enhanced CTL induction and antitumor effects of TAS0314.

According to the current study, immunization with TAS0314 and boosting 148 days after the last immunization significantly induced a large number of CTLs, suggesting that TAS0314 vaccination resulted in memory CTL induction. Among peripheral blood mononuclear cells, central memory T cells produce IL-2^35^, and autocrine IL-2 production is required for efficient expansion after a second challenge^36^. Therefore, multifunctional CTLs that are induced by TAS0314 might differentiate into memory T cells, and contribute to long-term surveillance. It has been reported that epitope-specific memory T cells remained for a long time in long-surviving patients treated with cancer vaccines^37^. Thus, TAS0314 might also contribute to the long-term control of cancer progression by inducing memory CTLs.

Cancer antigen-specific T cells upregulate immunosuppressive molecules (e.g., PD-1) after activation, and cause dysfunction by interacting with their ligands (e.g., PD-L1) in tumors^17^. Indeed, it has been reported that epitope-specific CTLs induced by peptide vaccines highly expressed PD-1 in cancer patients^38^. It is believed that the combination therapy of a cancer vaccine and PD-1/PD-L1 blockade synergistically enhances the antitumor activity. However, few HLA-A2-restricted epitope peptides from human cancer antigens have been evaluated for their efficacy in combination with anti-PD-1 antibody due to the HLA restriction in mouse models^39^. To evaluate the combination of other HLA allele-restricted epitopes with anti-PD-1 antibody, mice expressing the HLA allele are required. In the current study, we focused on the HLA-A24-restricted epitope, which is the most common HLA allele in Japanese. The synergistic antitumor effect of HLA-A24-restricted epitopes and anti-PD-1 antibody was evaluated using our HLA-A*2402 KI mice.

Combination treatment with TAS0314 and PD-1/PD-L1 blockade also showed synergistic antitumor activity. The combination therapy dramatically inhibited tumor growth, and prolonged survival. The number of epitope-specific CTLs in tumors were increased by approximately three-fold, and this increase was considered to be a mechanism of the synergistic antitumor effect of both PD-1 and PD-L1 antibody treatment with TAS0314.

Although a short peptide vaccine containing the SART2_93–101_ epitope did not induce a significant clinical response in a recent Phase III clinical trial, our data suggest that the combination of PD-1/PD-L1 blockade might improve its clinical efficacy.

It has been reported that anti-PD-1 antibody enhanced chemokine expression in tumors and T cell infiltration^24,40^. Others have also reported that anti-PD-1 antibody inhibited T cell apoptosis, and enhanced the proliferation of T cells^41^. According to these reports and our current data, we believe that anti-PD-1 antibody treatment induces the infiltration and accumulation of TAS0314-specific CTLs in the tumor microenvironment, which contributes to the synergistic antitumor effects. However, the detailed mechanism should be elucidated in future studies.

There are some limitations in this study. First, we evaluated the antitumor activity only against tumor cells expressing SART2_93–101_ as a representative epitope peptide. Other epitopes are also expected to show superior CTL induction and antitumor activity, because they likely behave similarly.

We also developed different multivalent long peptides, TAS0315 and TAS0316, which contain 8 epitopes from EGFR, Lck, MRP3, PTHrP, TMEM189, and WHSC2. Similar to TAS0314, these two long peptides also showed CTL induction ability in HLA-KI mice (Supplementary Fig. 1). According to our results, TAS0314, TAS0315, and TAS0316 are expected to have an antitumor effect and synergistic effect with anti-PD-1 antibody; however, further investigations are needed with the establishment of new HLA-A2- or A24-dependent syngeneic antitumor models.

Second, the antitumor effect of TAS0314 was examined only by an artificial model using HLA-A*2402 and SART2_93–101_ stably overexpressing cells. Although we confirmed that TAS0314-induced antigen-specific CTLs recognized and killed HLA-A24 human cancer cells in vitro (Supplementary Fig. 2), the anti-tumor effect of TAS0314 against human cancers remains to be clarified through clinical trials.

In summary, a novel multi-epitope long peptide vaccine, TAS0314, induced HLA-A2, HLA-A24*,* and HLA-A3 superfamily-restricted multiple epitope-specific CTLs. TAS0314 showed superior antitumor activity when compared to short epitope vaccines. Furthermore, we developed a combination model of vaccine and PD-1/PD-L1 blockade in HLA-A*2402 KI mice; combination therapy with anti-PD-1 antibody or anti-PD-L1 antibody increased epitope-specific CTLs in tumors and synergistically enhanced the antitumor activity. We have been developing a peptide mixture, TAS0313, that contains TAS0314, TAS0315, and TAS0316 for clinical use. Our preliminary data obtained during this development suggest that TAS0313 treatment, especially the combination therapy of TAS0313 and PD-1/PD-L1 blockade, represents a novel treatment option for patients suffering from various cancers. Moreover, the induced multiple antigen-specific CTLs that are expected to protect from immune escape due to antigen loss would contribute to long-term antitumor activity.

## Methods

### Mice

Heterozygous HLA-A*2402, heterozygous HLA-A*0201, and heterozygous HLA-A*3101 KI mice were generated as previously described^28^. All animal procedures were performed in compliance with the National Institutes of Health Guidelines, and were approved by the Taiho Institutional Animal Care and Use Committee.

### Peptide synthesis

TAS0314, TAS0315, TAS0316, and the epitope peptides in TAS0314 were synthesized and analyzed with liquid chromatography-mass spectrometry (LC–MS) by BACHEM Americas, Inc. (Torrance, CA, USA). HER2p63, WT1p126, and HIV gp41_770–780_ were synthesized and analyzed with LC–MS by Toray Research Center, Inc. (Tokyo, Japan). The peptide sequences are shown in Table 1.

**Table 1:** Amino acid sequences of TAS0314. CTL epitopes are shown in bold.

Name	Orientation	Epitope sequence
TAS0314	**SART2**_**93–101**_**-**RR**-SART3**_**734–743**_**-**RR**-SART3**_**109–118**_**-**RR**-SART3**_**302–310**_	**DYSARWNEI**RR**QIRPIFSNR**RR**VYDYNCHVDL**RR**LLQAEAPRL**
SART2_93–101_		**DYSARWNEI**
SART3_109–118_		**VYDYNCHVDL**
SART3_302–310_		**LLQAEAPRL**
SART3_734–743_		**QIRPIFSNR**
HER2p63		**TYLPTNASL**
HIV gp41_770–780_		**RLRDLLLIVTR**
WT1p126		**RMFPNAPYL**

### Antibodies and reagents

R-Phycoerythrin (PE)-conjugated anti-IFN-γ monoclonal antibody (mAb) (clone: XMG1.2), phycoerythrin-cyanin 7 (PE-Cy7)-conjugated anti-IL-2 mAb (clone: JES6-5H4), allophycocyanin (APC)-conjugated anti-TNF-α mAb (clone: MP6-XT22), Brilliant Violet (BV) 421 conjugated anti-CD8α mAb (clone: 53-6.7), BV 510-conjugated anti-CD90.2 mAb (clone: 30-H12), purified anti-HLA-ABC mAb (clone: w6/32), GoInVivo purified anti-mouse CD274 (PD-L1) mAb (clone: 10F.9G2), GoInVivo purified rat IgG2b, κ Isotype Ctrl Antibody (clone: RTK4530), and purified mouse IgG2a, κ Isotype Ctrl (clone: MOPC-173) were purchased from BioLegend, Inc. (San Diego, CA, USA). PE-conjugated HLA-A*24:02 SART2_93–101_ tetramer and fluorescein isothiocyanate (FITC)-conjugated anti-CD8 mAb (clone: KT15) were purchased from Medical and Biological Laboratories Co., Ltd. (Aichi, Japan). CD11c MicroBeads Ultrapure, mouse, Pan T Cell Isolation Kit II, mouse, CD90.2 MicroBeads, and Tumor Disassociation Kit, mouse, were purchased from Miltenyi Biotec (Bergisch Gladbach, Germany). Epoxomicin was purchased from Peptide Institute, Inc. (Osaka, Japan).

### Tumor cell lines

B16F10 and T2 were purchased from the American Type Culture Collection (ATCC, Manassas, VA, USA). B16F10.A24 cells and B16F10.A24/SART2_93–101_ cells were established by transducing the *HLA-A24* (HHD) gene or both the *HLA-A24* (HHD) gene and SART2_93–101_ mini-gene using the piggyBac vector (Transposagen Biopharmaceuticals, Inc., Lexington, KY, USA) as previously described^32^.

### Immunization of mice

Mice were immunized three times at 7-day intervals. A mixture of peptides emulsified in Montanide ISA-51VG (Seppic, Paris, France) was subcutaneously injected at the base of the tail (100 µL/mouse).

For the experiment to evaluate memory function, mice were immunized with TAS0314 or SART2_93–101_ [TAS0314: 100 µg/mouse, SART2_93–101_: 21 µg/mouse (equivalent molar mass to 100 µg of TAS0314/mouse)], and 148 days after the last immunization, the mice were immunized with SART2_93–101_ as a booster treatment. Two weeks after the booster treatment, CTL induction in the lymph node was evaluated.

### Culture of epitope-specific CTLs

Immunized mice were sacrificed 7 days after the last immunization, and lymphocytes from the lymph node were prepared. The lymphocytes were cultured for 8 days in culture medium supplemented with target peptide (10 µg/mL), rIL-15 (100 ng/mL) and mouse rIL-21 (100 ng/mL). Living cells were collected by density gradient centrifugation using Lympholyte-M (Cedarlane, Hornby, ON, Canada).

### Enzyme-linked immunospot (ELISPOT) assay

Epitope-specific IFN-γ production was detected using IFN-γ Plus ELISPOT (Mabtech, Stockholm, Sweden) as previously reported^32^. The lymphocytes from *HLA-KI* mice were cultured with the target epitope peptide (10 µg/mL) or negative control peptide (WT1p126 for HLA-A*0201; Her2p63 for HLA-A*2402; HIV-1 gp41_770–780_ for HLA-A*3101; all 10 µg/mL) in culture medium (RPMI-1640 containing 10% heat-inactivated fetal bovine serum, 100 U/mL penicillin, 100 µg/mL streptomycin, and 50 µM 2-mercaptoethanol). For cases in which epitope-specific IFN-γ production was detected in the cultured CTLs, the CTLs were subsequently cultured with irradiated (30 Gy) splenocytes and the indicated peptide (100 µg/mL) in culture medium. Detection of IFN-γ production was performed according to the Mabtech protocol. Then, IFN-γ spots were counted with an ELISPOT analyzer Immunospot S6 (Cellular Technology Ltd., Shaker Heights, OH, USA).

### Antigen presentation assay

CD11c^+^ dendritic cells (DCs) and CD3^+^ T cells were purified with CD11c MicroBeads UltraPure, mouse, and the Pan T Cell Isolation Kit II, mouse (Miltenyi Biotec) from the splenocytes of HLA-A*0201 KI mice. The antigen-presenting cells were incubated with peptides for 3 h (37 °C, 5% CO_2_) and washed three times. Epoxomicin was added 30 min before the addition of TAS0314. Cultured SART3_302–310_-specific CTLs from immunized HLA-A*0201 KI mice with SART3_302–310_ (100 μg/mice) were used as effector cells. The CTLs were co-cultivated with antigen-presenting cells on an anti-mouse IFN-γ-coated plate. Epitope-specific IFN-γ production was detected using an ELISPOT assay.

### Evaluation of antitumor activity against SART2_93–101_-expressing syngeneic tumors

For the prophylactic treatment, HLA-A*2402 KI mice (n = 10) were immunized three times with TAS0314 (100 µg/mouse), SART2_93–101_ epitope (21 µg/mouse), or vehicle control. Seven days after the last immunization, immunized mice were subcutaneously inoculated with B16F10.A24/SART2_93–101_ tumor cells (5 × 10^5^) into the right flank. Seven days after tumor inoculation, the tumor size was measured with a caliper. The tumor volume was calculated using the following formula:

$${\text{Tumor}}\;{\text{volume}}\;({\text{mm}}^{3} ) = {\text{length}}\;({\text{mm}}) \times [{\text{width}}\;({\text{mm}})]^{2} /2.$$

For the therapeutic treatment, HLA-A*2402 KI mice (n = 15) were intravenously injected with B16F10.A24/SART2_93–101_ tumor cells (1 × 10^6^). The mice were immunized with TAS0314 (300 µg/mouse) three times with intervals of 7 days starting from the day after the tumor inoculation. On day 28 after tumor injection, mice were euthanized, and the number of lung metastases was counted.

### Combination therapy of TAS0314 and anti-PD-1/PD-L1 antibody

To evaluate the efficacy of the combination therapy of TAS0314 and anti-PD-1/PD-L1 antibody, HLA-A*2402 KI mice (n = 15) were immunized three times with TAS0314 vaccine (300 µg/mouse) or vehicle control. Seven days after the last immunization, anti-PD-1 mAb (100 µg/mouse), anti-PD-L1 mAb (200 µg/mouse), or isotype rat IgG2b (200 µg/mouse) was injected intraperitoneally, and then B16F10.A24/SART2_93–101_ tumor cells were subcutaneously inoculated (5 × 10^5^) into the right flank of the mice.

### ^51^Cr cytotoxicity assay

The cytotoxicity of cultured CTLs against B16F10.A24 cells as well as SART2_93–101_ peptide-pulsed B16F10.A24 cells or B16F10.A24/SART2_93–101_ cells was evaluated with a 6h ^51^Cr-release assay as reported previously^42^. Cultured SART2_93–101_ epitope-specific CTLs from TAS0314-immunized mice (100 µg/immunization) were used as effector cells. ^51^Cr (5 MBq/10^6^ cells) and peptide (100 μmol/L) were added to the target cells and incubated for 1.5 h at 37 °C. After incubation, the target cells were washed with phosphate-buffered saline, and were suspended in culture medium. Five thousand ^51^Cr-labeled target cells were cultured with cultured CTLs in 96-well round bottom plates at the indicated effector/target ratios in triplicate assays.

The cytotoxicity of cultured CTLs against T2 cells as well as SART3_302–310_ peptide-pulsed T2 cells was evaluated using cultured SART3_302–310_ epitope-specific CTLs from TAS0314-immunized mice (300 µg/immunization) as effector cells.

Based on the measurement of radioactivity (cpm) in the supernatant, the percent specific lysis was calculated by the following formula:

$$\% \;{\text{specific}}\;{\text{lysis}} = [{\text{test}}\;^{51} {\text{Cr}}\;{\text{release}}\;({\text{cpm}}) - {\text{mean}}\;{\text{spontaneous}}\;^{51} {\text{Cr}}\;{\text{release}}\;({\text{cpm}})]/[{\text{mean}}\;{\text{maximal}}\;^{51} {\text{Cr}}\;{\text{release}}\;({\text{cpm}}) - {\text{mean}}\;{\text{spontaneous}}\;^{51} {\text{Cr}}\;{\text{release}}\;({\text{cpm}}) ]\times 100.$$

### Isolation of tumor-infiltrating lymphocytes

Single-cell suspensions from tumors were prepared using the Tumor Disassociation Kit, mouse, and GentleMACS (Miltenyi Biotec) according to the manufacturer’s procedures. Living lymphocytes were isolated followed by density gradient centrifugation on a 70%/40% Percoll (GE Healthcare, Chicago, IL, USA) gradient. After density gradient centrifugation, T cells were isolated with CD90.2 MicroBeads and cultured overnight in culture medium. The cultured T cells were stained with SART2_93–101_ tetramer, anti-CD8-APC mAb (clone KT15), and anti-CD90.2-BV510 mAb. CD90.2^+^CD8^+^ cells were analyzed by FACSVerse (BD Bioscience, San Jose, CA, USA).

### Detection of cytokine production by CD8^+^ T cells

To evaluate cytokine multi-functionality, whole blood from immunized HLA-A*2402 KI mice was lysed with BD Pharm Lyse (BD Bioscience), stimulated with 10 µg/mL SART2_93–101_ peptide, and cultured for 18 h supplemented with Golgiplug (BD Bioscience). Then, stimulated peripheral blood mononuclear cells were stained with anti-CD8-BV421 mAb and anti-CD90.2-BV510 mAb. The cells were fixed and permeabilized using the Fixation/Permeabilization Solution Kit (BD Bioscience), then stained with anti-IFN-γ-PE mAb, anti-TNF-α-APC mAb, and anti-IL-2-PE-Cy7 mAb. Stained cells were analyzed by FACSVerse.

### Statistical analysis

The statistical significance of CTL induction, tumor volume, and lung metastasis was evaluated with a two-tailed Student’s *t* test. The statistical significance of survival was evaluated with a log-rank test. In all analyses, *P* < 0.05 was considered to indicate statistical significance.

## Supplementary information

Supplementary Information.

## Data Availability

The datasets generated and/or analyzed in the current study are available from the corresponding author on reasonable request.
