# Author Correction: TAS0314, a novel multi-epitope long peptide vaccine, showed synergistic antitumor immunity with PD-1/PD-L1 blockade in HLA-A*2402 mice

**DOI:** 10.1038/s41598-022-10374-x

**Published:** 2022-04-12

**Authors:** Yuki Tanaka, Hiroshi Wada, Risa Goto, Toshihiro Osada, Keisuke Yamamura, Satoshi Fukaya, Atsushi Shimizu, Mitsuru Okubo, Kazuhisa Minamiguchi, Koichi Ikizawa, Eiji Sasaki, Teruhiro Utsugi

**Affiliations:** Discovery and Preclinical Research Division, Taiho Pharmaceutical Co. Ltd., Tsukuba, Ibaraki Japan

Correction to: *Scientific Reports* 10.1038/s41598-020-74187-6, published online 14 October 2020

The original version of this Article contained an error in Figure 2 where the average spot for “CD11c^+^ DC + Epoxomicin” was incorrect in panel (d). The original Figure 2 and accompanying legend appear below.

**Figure 2:**
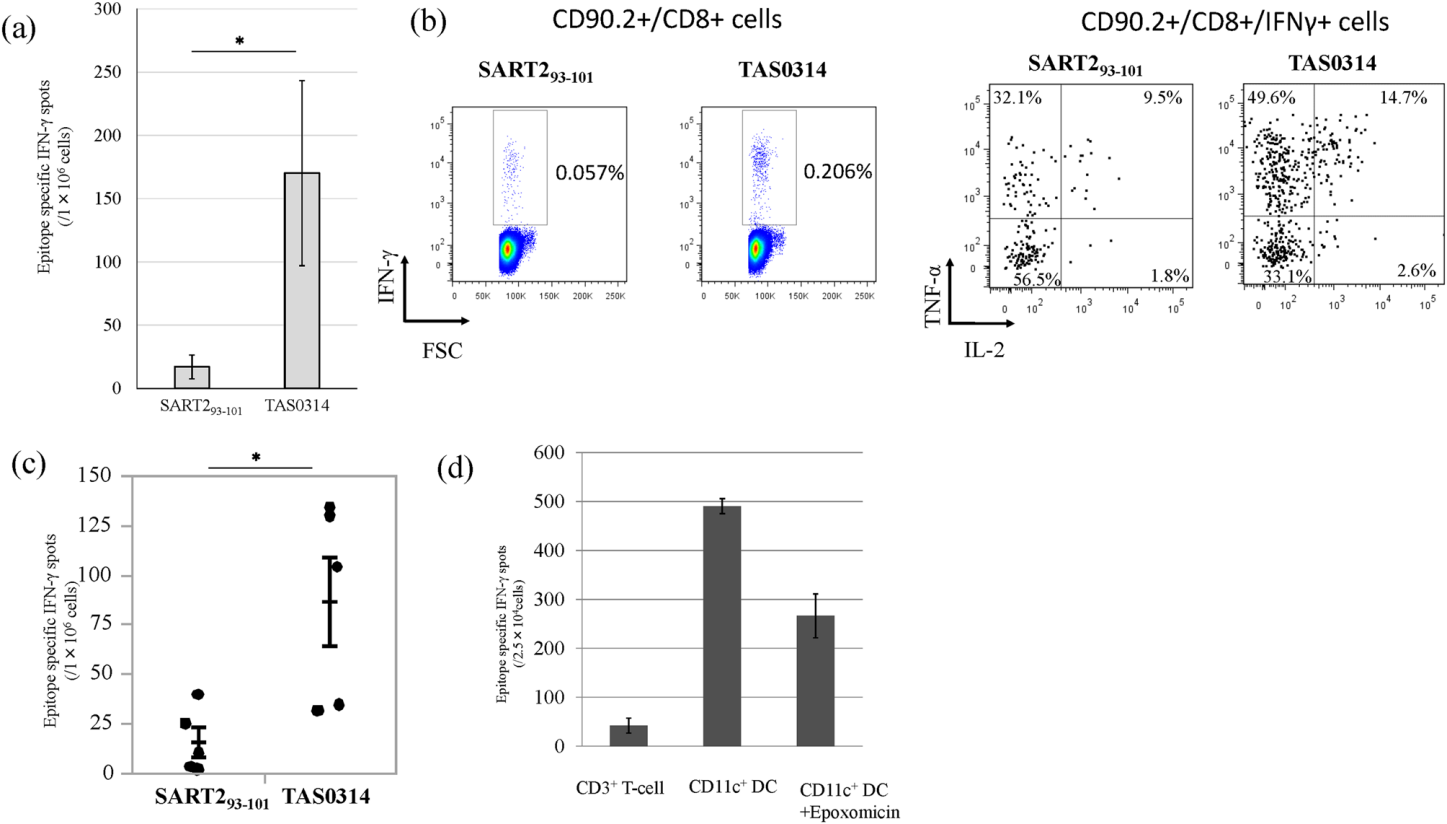
Comparison of the SART2_93–101_-specific CTL induction and its function between short (SART2_93–101_) and long (TAS0314) peptides. (**a**) Comparison of the frequency of SART2_93–101_-specific CTLs. HLA-*A2402 KI mice (n = 10/group) were vaccinated with TAS0314 (100 µg) or SART2_93–101_ peptide (21 µg, equivalent molar mass to TAS0314). One week after the last immunization, SART2_93–101_-specific IFN-γ production from the lymphocytes of each mouse was evaluated with an IFN-γ ELISPOT assay. Data represent the mean ± standard error (n = 10). (**b**) Cytokine multi-functionality of SART2_93–101_-specific CTLs. Peripheral blood mononuclear cells were prepared from immunized mice (n = 10/group) and stimulated overnight with SART2_93–101_ peptide (10 μM). The production of IFN-γ, TNF-α, and IL-2 was analyzed in CD90.2^+^/CD8^+^ cells. (**c**) Comparison of the frequency of SART2_93–101_-specific CTLs in the prime-boost vaccination condition. HLA-*A2402 KI mice (n = 5/group) were vaccinated three times at weekly intervals with TAS0314 (100 µg) or SART2_93–101_ peptide (21 µg). One hundred and forty-eight days after the last immunization, all mice were immunized with SART2_93–101_ peptide (21 µg). Data represent the mean ± standard error (n = 5). (**d**) Evaluation of the antigen presentation of TAS0314. SART3_302–310_ epitope-specific CTLs were cultured with TAS0314-pulsed CD3^+^ T cells, CD11c^+^ DCs, or epoxomicin-treated CD11c^+^ DCs. Data represent the mean ± standard deviation (n = 4). *p < 0.05 using a two-tailed Student’s *t* test.

The original Article has been corrected.

