# Supplementary material for: TAS0314, a novel multi-epitope long peptide vaccine, showed synergistic antitumor immunity with PD-1/PD-L1 blockade in HLA-A*2402 mice
Source: Sci Rep. 2020 Oct 14;10:17284. doi: 10.1038/s41598-020-74187-6 (PMC7560884; doi:10.1038/s41598-020-74187-6)
Supplement: Supplementary file 1 — Supplementary Information. [file 41598_2020_74187_MOESM1_ESM.pdf]

TAS0314, a novel multi-epitope long peptide vaccine, showed synergistic antitumor immunity with PD-1/PD-L1 blockade in HLA-A*2402 mice.

Yuki Tanaka[1],*, Hiroshi Wada[1], Risa Goto[1], Toshihiro Osada[1], Keisuke Yamamura[1], Satoshi Fukaya[1], Atsushi Shimizu[1], Mitsuru Okubo[1], Kazuhisa Minamiguchi[1], Koichi Ikizawa[1], Eiji Sasaki[1] and Teruhiro Utsugi[1]

[1] Discovery and Preclinical Research Division, Taiho Pharmaceutical Co. Ltd., Tsukuba, Ibaraki, Japan

Corresponding Author:

*Yuki Tanaka

Email: yuuki-tanaka@taiho.co.jp

Name	Orientation	Epitope Sequence
**TAS0315**	**EGFR**$_{800-809}$-RR-**PTHrP**$_{102-111}$-RR-**Lck**$_{246-254}$-RR-**Lck**$_{90-99}$	**DYVREHKDNI**RR**RYLTQETNKV**RR**KLVERLGAA**RR**ILEQSGEWWK**
**TAS0316**	RRRR-**MRP3**$_{503-511}$-RR-**TMEM189**$_{43-51}$-RR-**Lck**$_{488-497}$-RR-**WHSC2**$_{103-111}$	RRRR**LYAWEPSFL**RR**RLQEWCSVI**RR**DYLRSVLEDF**RR**ASLDSDPWV**
**EGFR**$_{800-809}$		**DYVREHKDNI**
**Lck**$_{90-99}$		**ILEQSGEWWK**
**Lck**$_{246-254}$		**KLVERLGAA**
**Lck**$_{488-497}$		**DYLRSVLEDF**
**MRP3**$_{503-511}$		**LYAWEPSFL**
**PTHrP**$_{102-111}$		**RYLTQETNKV**
**TMEM189**$_{43-51}$		**RLQEWCSV**
**WHSC2**$_{103-111}$		**ASLDSDPWV**

Supplementary Table. 1 Amino acid sequences of TAS0315 and TAS0316.

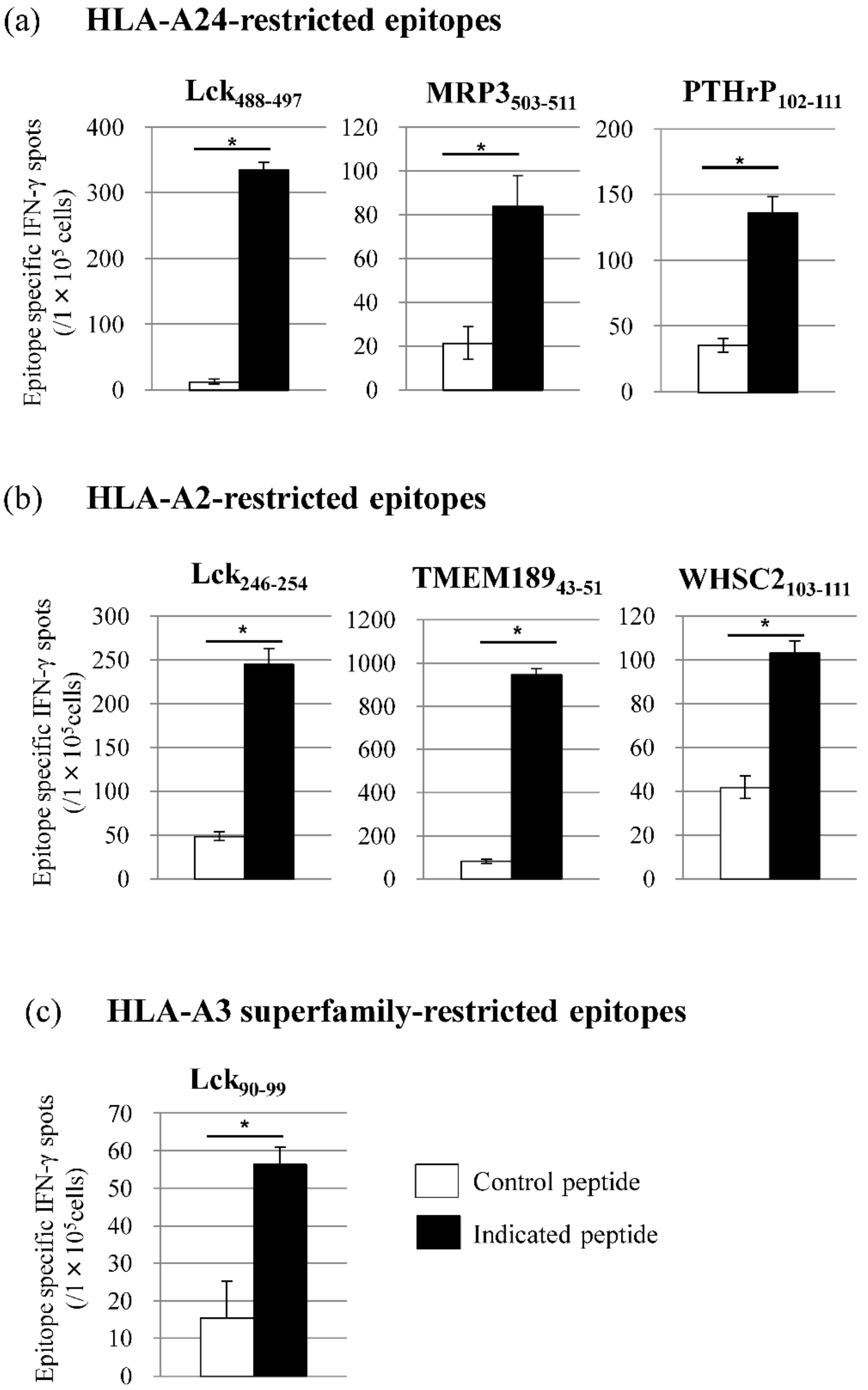

Supplementary Figure. 1 CTL responses were induced by TAS0313 vaccination (300 μg/each peptide) in various HLA-KI mice (n = 10). One week after the last immunization,

draining lymph node cells were isolated and cultured for eight days with epitope peptide, IL-15 and IL-21.Epitope-specific CTLs were evaluated with an IFN-γ ELISPOT assay. Data represented mean ± s.d. (n = 4).

(a) HLA-A24-restricted epitope-specific CTL induction in *HLA-A24* KI mice
(b) HLA-A2-restricted epitope-specific CTL induction in *HLA-A2* KI mice
(c) HLA-A3 superfamily-restricted epitope-specific CTL induction in *HLA-A31* KI mice

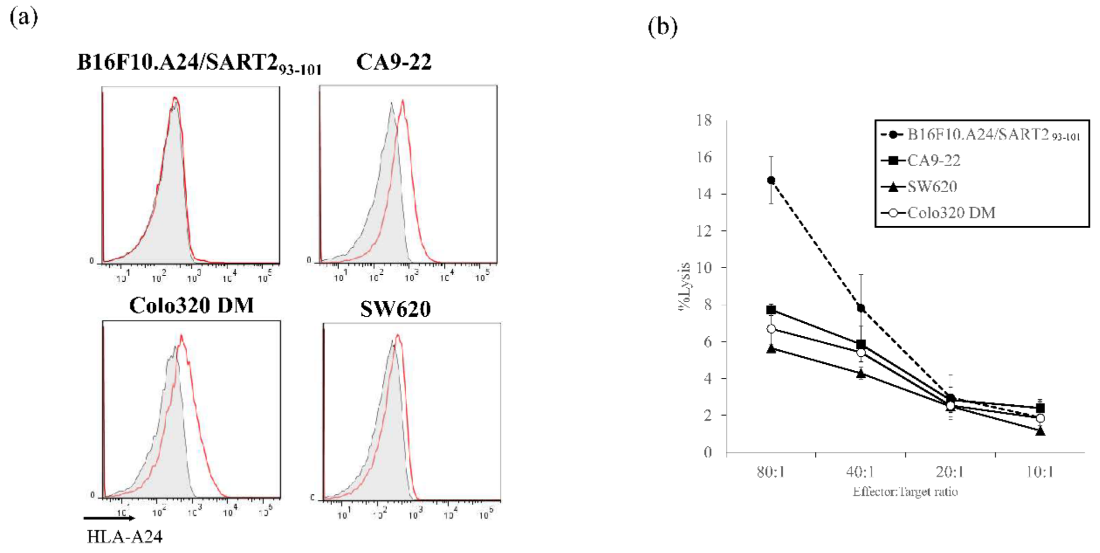

Supplementary Figure. 2 (a) Comparison of HLA-A24 expression. B16F10.A24/SART2$_{93-101}$, CA9-22, Colo320 DM and SW620 were stained with anti-HLA-A24 antibody-PE (Red line, clone:17A10, Medical & Biological Laboratories Co., Ltd.) or PE Mouse IgG2b (gray line with shadow, MG2b-57, BioLegend, Inc.). Stained cells were analyzed by CytoFlex (Beckman Coulter Inc).
(b) The cytotoxicity of cultured CTLs against B16F10.A24 cells and Human cancer cell lines. Data represent the mean ± standard deviation (n = 3).

Supplemental Information

Peptide synthesis

TAS0315, TAS0316, and the epitope peptides in TAS0315 and TAS0316 were synthesized and analyzed with liquid chromatography-mass spectrometry (LC-MS) by BACHEM Americas, Inc. (Torrance, CA, USA). HER2p63, WT1p126, and HIV gp41770-780 were synthesized and analyzed with LC-MS by Toray Research Center, Inc. (Tokyo, Japan).

Tumor cell lines

SW620 (CCL-227) were purchased from ATCC. CA9-22 (JCRB0625, [Studies on lactate dehydrogenase isoenzymes in a cell line (Ca 9-22) derived from carcinoma of the gingiva (author's transl)].Kimura YKokubyo Gakkai Zasshi. 1978 Mar;45(1):20-35) and Colo320 DM(JCRB0225, Cell lines from human colon carcinoma with unusual cell products, double minutes, and homogeneously staining regions.Quinn LA,Moore GE,Morgan RT,Woods LK Cancer Res. 1979 Dec;39(12):4914-24) were obtained from Japanese Collection of Research Bioresources Cell Bank (Osaka, Japan).
